# Type 1 diabetes incidence during COVID-19 pandemic has not been influenced by COVID-19 vaccination in northern Italy region, Lombardy

**DOI:** 10.1371/journal.pone.0316340

**Published:** 2025-02-10

**Authors:** Chiara Mameli, Camilla Valsecchi, Danilo Cereda, Simona Scarioni, Lucia Barcellini, Catia Boriello, Riccardo Bonfanti, Ilaria Brambilla, Valeria Calcaterra, Elena Calzi, Roberta Cardani, Anna Cogliardi, Barbara Felappi, Giorgia Florini, Giulio Frontino, Lucia Paola Guerraggio, Adelina Hajro, Maddalena Macedoni, Patrizia Macellaro, Agnese Petitti, Carmelo Pistone, Cristina Provero, Silvia Radaelli, Francesca Chiara Redaelli, Andrea Rigamonti, Andrea Scaramuzza, Silvia Sordelli, Daniele Spiri, Ciretta Pelliccia, Maria Zampolli, Gian Vincenzo Zuccotti

**Affiliations:** 1 Department of Pediatrics, V Buzzi Children’s Hospital, Università di Milano, Milan, Italy; 2 Department of Biomedical and Clinical Science, Università di Milano, Milan, Italy; 3 Direzione Generale (DG) Welfare, Milan, Lombardy Region, Italy; 4 Department Biomedical Sciences for Health, Postgraduate School in Public Health, Università di Milano, Milan, Italy; 5 Pediatric Diabetes, Diabetes Research Institute, Scientific San Raffaele Hospital and Vita Salute San Raffaele University, Milan, Italy; 6 Department of Pediatrics, IRCCS Policlinico San Matteo, Pavia, Italy; 7 Department of Internal Medicine, University of Pavia, Pavia, Italy; 8 Department of Pediatrics, Ospedale Maggiore, ASST Crema, Crema, Italy; 9 Department of Pediatrics, Ospedale Del Ponte Varese, ASST Sette Laghi, Varese, Italy; 10 Department of Pediatrics, Ospedale Alessandro Manzoni, Lecco, Italy; 11 Department of Pediatrics, ASST Spedali Civili, Brescia, Italy; 12 Department of Pediatrics, Ospedale di Tradate, ASST Sette Laghi, Tradate, Italy; 13 Department of Pediatrics, Ospedale di Legnano, ASST Ovest milanese, Legnano, Italy; 14 Department of Pediatrics, ASST Papa Giovanni XXIII, Bergamo, Italy; 15 Department of Pediatrics, Ospedale Maggiore, ASST Cremona, Cremona, Italy; 16 Department of Pediatrics, Ospedale Carlo Poma, Mantova, Italy; 17 Department of Pediatrics, Ospedale Sant’Anna, Como, Italy; CNR, National Research Council of Italy, ITALY

## Abstract

**Objective:**

To describe the trends of type 1 diabetes(T1D) incidence in 0–17-year-olds over the years 2020–2023, and the COVID-19 vaccination uptake in Lombardy region.

**Methods:**

Data about children and adolescents aged 0–17 years who received a diagnosis of T1D from 2020 to 2023 were extracted from the public computerized registry of the healthcare system of the Lombardy Region (Italy). After calculating the annual T1D incidence, the incidence in 2020, prior to the availability of vaccination, was compared to subsequent years. A separate analysis was conducted for the 12–17 age group, the first to receive vaccination.

**Results:**

One thousand two hundred seventy-three T1D onsets were recorded. The distribution of T1D showed no significant annual variation by sex (p-trend = 0.338), mean age (9 years, p = 0.537) and age distribution (p-trend = 0.563). T1D incidence [95% CI/100.000] did not significantly change comparing 2020 [18.94/100.000 (CI 16.88–21.18)] with 2021 [21.82/100.000 (CI 18.90–23.44)], 2022 [20.77/100.000 (CI 18.59–23.13)] and 2023 [19.68/100.000 (CI 16.61–20.94)]. No differences in incidence were observed in the 12–17 age group during 2021–2023 when COVID-19 vaccination was available when compared to 2020 (p-wald > 0.05). The COVID-19 vaccination coverage was lower in children with diabetes onset compared to the same-age general population (38 vs 42%).

**Conclusions:**

The incidence of T1D in children remained stable during the COVID-19 pandemic, regardless of the uptake of the vaccination.

## 1. Introduction

The incidence of type 1 diabetes (T1D) has been increasing in developed countries over the last 30 years, especially in Europe [1]. Several studies have reported an increasing incidence of T1D onset during COVID-19 pandemic in paediatric population [2–5]. The incidence rate is higher, but not significantly, compared with the pre-pandemic period in most studies [2]. Therefore, it is unknown whether the incidence of T1D during the pandemic period was entirely influenced by COVID-19 or whether the reported increasing incidence is a part of the global trend of the disease [2,6].

In Europe, the Lombardy Region in Northern Italy experienced one of the most massive and deadliest COVID-19 outbreaks, offering a unique epidemiologic scenario over the years 2020–2023 during the pandemic years. The COVID-19 vaccination campaign in Italy was a mass immunization carried out by the Italian government starting at the end of 2020 in at-risk populations and spreading to all the population in the following months. COVID-19 vaccines have been actively offered to teenagers 12–17 years since June 2021; children from 5 to 11 years old could universally get the vaccination at the beginning of 2023 [7]. With the widespread of COVID-19 infection and COVID-19 vaccines, many complications have appeared, including autoimmune diseases. Some authors suggested the potential roles of COVID-19 infection and vaccine-derived antigens in autoimmune disease development. However, evidence is conflicting and inconclusive [8,9]. We used regional aggregated data to describe the trends of T1D incidence in 0–17-year olds over the years 2020–2023 and the COVID-19 vaccination uptake in the Lombardy region.

## 2. Materials and methods

### 2.1. Data sources

To estimate the incidence of paediatric T1Dduring the calendar years 2020–2023, we used 2 data sources: the Lombardy Health Care System database and the demographic data of the Lombardy Region retrieved from the page (http://demo.istat.it/) referencing the National Institute of Statistics (ISTAT). The Lombardy Health Care System database is a public computerized registry that includes health-related anonymized data recorded in the Lombardy Region. The available ISTAT information consists of the amount of the resident population in Lombardy on January 1^st^ for each year of interest, also stratified by gender and age.

The Lombardy Health Care System database was used to estimate the Covid-19 vaccine uptake in T1D onset and the general population. A person who had received at least one COVID-19 vaccine dose was considered vaccinated.

### 2.2. Procedures

From 3^rd^ to 30^th^ January 2024, data about subjects between 0 and 17 years who had received a diagnosis of T1D from 2020 to 2023 in the Lombardy Region were extracted from the Lombardy Health Care System database by using

- Hospital discharge from 2020 to 2023 with International Classification of Diseases, Ninth Revision, Clinical Modification (ICD-9-CM code 250.01, 250.03, 250.11, 250.13, 250.21, 250.23, 250.31, or 250.33) as indicated in the Hospital Discharge Form;
- Hospitalization with Diagnosis-Related Group (DRG) 295 as indicated in the Hospital Discharge Form.

For each hospitalization record, anonymized data relating to the patient’s age and sex along with the date of each diagnosis, were collected. Data relating to the type of vaccine received, the date of vaccination and number of doses were also extracted. Authors had no information to identify individual participants during or after data collection.

The outcomes were: to describe the yearly incidence of clinically diagnosed paediatric T1D for the years 2020–2023 and to compare COVID-19 vaccination uptake in subjects who had T1D onset with that in the same-age paediatric population (0–17 years) in Lombardy.

T1D was diagnosed according to the guidelines of the International Society for Pediatric and Adolescent Diabetes [10]. Subjects with non-T1D and new-onset diabetes of unclear type were excluded.

### 2.3. Covariates and statistical analysis

The analyzed study participants’ characteristics included the diabetes diagnosis date, sex, age (continuous and categorical), COVID-19 vaccination uptake, and the number of vaccine doses received.

The characteristics of T1D subjects were summarized as medians and interquartile ranges for continuous variables and as frequencies for categorical variables, overall and for each year from 2020 to 2023. The incidence proportions for 2020 to 2023 were estimated using generalized regression models with a Poisson distribution, with new onsets as the dependent variable, year as the independent variable, and population size as the offset. All incidence proportions were expressed as the number of incident cases per 100,000 subjects in the population.

Next, incidence rate ratios (IRRs) and 95% confidence intervals (CIs) were calculated to examine relative differences in T1D incidence during 2020 compared with the subsequent years (2021–2023), overall and by age group.

Analyses were performed using Stata version 18.

## 3. Results

From the data retrieved, we registered 1273 cases of new-onset paediatric subjects with T1D aged 0–17 years, from 2020–2023. The distribution of T1D onset showed no significant annual variation by sex (p trend = 0.338), mean age (9 years, p = 0.537), age group distribution (p trend = 0.563) (Table 1).

**Table 1:** Characteristics of incident paediatric type 1 diabetes, 2020–2023.

	Total n°	2020 n° (%)	2021 n° (%)	2022 n° (%)	2023 n° (%)	P value
Type 1 diabetes						
N° of observation	1,273	308 (24.19)	340 (26.71)	331 (26.00)	294 (23.10)	
Sex					
Male	695	173 (24.89)	196 (28.20)	176 (25.32)	150 (21.58)	0.338
Female	578	135 (23.36)	144 (24.91)	155 (26.82)	144 (24.91)	
Median age (IQR)^a^	9 (6–13)	10 (6–12)	9 (6–12)	10 (6–13)	9 (6–13)	0.537
Age category					
0–4 years	208	59 (28.37)	55 (26.44)	51 (24.52)	43 (20.67)	0.563
5–11 years	636	156 (24.53)	171 (26.89)	168 (26.42)	141 (22.17)	
12–17 years	429	93 (21.68)	114 (26.57)	112 (26.11)	110 (25.64)	
COVID-19 vaccinated	293	0	38 (12.97)	149 (50.85)	106 (36.18)	<0.001
Provinces						0.145
Bergamo	158	42 (26.58)	47 (29.75)	36 (22.78)	33 (20.89)	
Brescia	179	39 (21.79)	56 (31.28)	43 (24.02)	41 (22.91)	
Como	68	15 (22.06)	22 (32.35)	11 (16.18)	20 (29.41)	
Cremona	46	14 (30.43)	11 (23.91)	10 (21.74)	11 (23.91)	
Lecco	39	9 (23.08)	15 (38.46)	7 (17.95)	8 (20.51)	
Lodi	21	8 (38.10)	5 (23.81)	7 (33.33)	1 (4.76)	
Mantova	51	7 (13.73)	13 (25.49)	17 (33.33)	14 (27.45)	
Milano	344	90 (26.16)	81 (23.55)	88 (25.58)	85 (24.71)	
Monza	94	25 (26.60)	20 (21.28)	30 (31.91)	19 (20.21)	
Pavia	60	15 (25.00)	15 (25.00)	23 (38.33)	7 (11.67)	
Sondrio	23	4 (17.39)	8 (34.78)	7 (30.43)	4 (17.39)	
Varese	123	25 (20.33)	31 (25.20)	32 (26.02)	35 (28.46)	
Not available	20	3 (15.00)	2 (10.00)	10 (50.00)	5 (25.00)	

The last column shows the results from the comparison tests throughout the four years: ages median was compared by the Kruskal-Wallis test; for any other variables, the comparisons were performed by test for trends.

^a^interquantile range

### 3.1. Epidemiology of type 1 diabetes from 2020 to 2023

Overall, T1D onsets were 308 in 2020, 340 in 2021, 331 in 2022, and 294 in 2023 (Table 1). The incidence [95% CI/100,000 persons] was 18.94/100.000 (CI 16.88–21.18) in the calendar year 2020, compared to 21.08 (CI 18.90–23.44), 20.77 (CI 18.59–23.13) and 18.68 (CI 16.61–20.94), respectively for the years 2021, 2022 and 2023 (Table 2). It emerged that the incidence of T1D did not change from the start to the end of the COVID-19 pandemic, even if a slight increase in T1D incidence in 2021 and 2022 was observed.

**Table 2:** Incidence of type 1 diabetes over the years 2020, 2021, 2022 and 2023 by Poisson regression models. Incidence estimates were expressed as per 100,000 subjects in the target population (0–17 years).

Column1	cases	pop at risk	incidence x 100.000 p	cIR^a^	95% CI^b^	p wald
**year**						
**2020**	308	1626098	18.94 (16.88–21.18)	ref		
**2021**	340	1612906	21.08 (18.90–23.44)	1.11	0.954–1.298	0.174
**2022**	331	1593740	20.77 (18.59–23.13)	1.10	0.939–1.286	0.245
**2023**	294	1573871	18.68 (16.61–20.94)	0.99	0.841–1.157	0.865

^a^cIR crude incidence ratio.

^b^confidence interval.

### 3.2. COVID-19 vaccination uptake among type 1 diabetes onset and the general pediatric population

The incidence of T1D was assessed separately for age class 12–17 years, as it was the only one accessing COVID-19 vaccination from 2021. The incidence observed in the 12–17 age group in the years 2021, 22, 23 when COVID-19 vaccination is similar to that registered in 2020 (p wald > 0.05) (Table 3).

**Table 3:** Incidence of type 1 diabetes over the years 2020, 2021, 2022 and 2023 by Poisson regression models in adolescents aged 12–17 years. Estimates of incidence were expressed as per 100,000 subjects in the target population.

Column1	cases	pop at risk	incidence x 100.000 p	cIR^a^	95% CI	p wald
**year**						
**2020**	93	57279	16.19 (13.07–19.84)	ref		
**2021**	114	582178	19.58 (16.15–23.52)	1.21	0.919–1.590	0.174
**2022**	112	590516	18.97 (15.62–22.82)	1.17	0.890–1.542	0.260
**2023**	110	593799	18.52 (15.23–22.33)	1.14	0.868–1.508	0.340

^a^cIR crude incidence ratio

In Lombardy, children with T1D onset aged 12–17 years, the only age group that benefited from vaccination in 2023, were slightly less likely to be vaccinated against COVID-19 than the general paediatric population. The vaccination uptake for this group was 67.3% (95%CI 57.7–76.0), while the vaccination uptake for the general paediatric population was 74.7% (95% CI 74.6–74.8) (p-value t-test 0.07) (Table 4).

**Table 4:** COVID-19 vaccination uptake among type 1 diabetes subjects and the general pediatric population in the Lombardy region in 2023.

	Vaccinated n°	Total population n°	Vaccine uptake % (95% CI^a^)
**Type 1 diabetes (0–17)**	106	294	39.5% (33.8–45.3)
**age, years (y)**			
**0–4 y**	0	43	0% (0–8.2)
**5–11 y**	32	141	22.7% (16.1–30.6)
**12–17 y**	74	110	67.3% (57.7–76.0)
**general population (0–17)**	705863	1665160	42.4% (42.3–42.5)
**age, years**			
**0–4 y**	262	350011	0.0% (0.0–0.0)
**5–11 y**	177688	608334	29.2% (29.1–29.3)
**12–17 y**	527913	706815	74.7% (74.6–74.8)

^a^Confidence interval.

The COVID-19 vaccination uptake in 0–17 years was 39.5% (95%CI 33.8–45.3) in T1D onset vs 42.4% 95% CI (42.3–42.5) in the general population (p-value T-test = 0.31).

## 4. Discussion

In the present study, we observed that the incidence of T1D in children aged 0–17 years remained stable during the COVID-19 pandemic in the Lombardy region, Northern Italy, one of the most severely affected regions in Europe, even if a slight increase in T1D incidence in children was seen in 2021 and 2022. The distribution of cases showed no significant annual variation by sex, mean age and age group distribution. In 2022, an article from the U.S. Centers for Disease Control and Prevention reported, based on medical claims databases, increased incidence of diabetes after COVID-19 infection among those younger than 18 years [11]. Such a finding would imply a substantial increase in the burden of childhood T1D. However, similar estimates have not been replicated [12].

To the best of our knowledge, this is the first study reporting the incidence from the start to the end of the COVID-19 pandemic. The studies published so far were conducted during the first pandemic wave. No data are currently available during the late pandemic [2]. According to the available data, in Lombardy the incidence of T1D showed an increase from 7/100.000 person in children aged 0–14 years in 1989 to 14/100.000 in 2019 (pre-covid era, 0–17 years) and reaching 21/100.000 in 2021 in children aged 0–17 years [13–15]. Even if the incidence in 1989 did not refer to the same age population, our data suggests a worrying upward trend as reported in most European countries [16,17].

The COVID-19 vaccine effectively reduced the impact of COVID-19 outbreak in northern Italy [18]. Children and adolescents aged over 12 years were the first to be vaccinated in Lombardy in 2021, shortly after the regulation agencies’ approval. We provide a descriptive analysis of T1D incidence trends and COVID-19 vaccination uptake in the age group 12–17 years, which was the age group who had accessed the vaccination since the beginning of the national immunization campaign. The incidence of T1D in the 12–17 age group has not shown substantial changes over the period 2020–2023, despite the availability of COVID-19 vaccination. However, since vaccine uptake was suboptimal in both the diabetic and general populations, even if vaccination were to influence diabetes incidence, it would be difficult to observe this effect in time trend analyses.

Moreover, we compared the COVID-19 vaccination uptake among T1D onset to the general population in Lombardy. We observed that at the onset of the disease, children who developed T1D were less likely to be vaccinated than the general population, despite equal opportunities for vaccination. We can speculate that in the Lombardy Region, diabetes affected racial, ethnic minority, and low-income populations disproportionately, as reported in the U.S. adult population, especially during the COVID-19 pandemic [19]. Due to social, financial and educational competition, this population could infrequently interface with health care and may miss critical opportunities to prevent COVID-19. Unfortunately, the determinants that could have led to reduced access to health care and social support, such as low income, low parental education levels, poor living conditions, and ethnicity, cannot be extracted from the Lombardy Health Care System database. These factors could have affected both vaccination uptake and diabetes diagnosis rates. Further research will be warranted to explore the reasons for their reduced access to preventive healthcare measures.

Our results suggested that the incidence of T1D in children and adolescents aged 0–17 remained stable during the pandemic period, regardless of Covid vaccination uptake. Covid-19 vaccination did not increase the incidence of T1D as previously suggested by other authors in both adult and paediatric populations [20]. A putative link between the SARS-CoV-2 infection and T1D onset as well as COVID-19 vaccine administration and T1D onset were suggested. It was speculated that the COVID-19 vaccine may trigger an immunological response that may lead to an overproduction of pro-inflammatory cytokines, like the SARS-CoV-2 infection, exacerbating pancreatic beta-cell inflammation and contributing to clinically manifest diabetes [21,22]. On the contrary, although our data reflect only descriptive trends in incidence, are in line with the most recent epidemiological data and seem not to support such an association [23].

Our study has some limitations. First, the aggregated data allowed only a descriptive analysis of diabetes incidence time trends. The study is not designed nor powered to conduct a causal interference analysis of the effect of COVID-19 vaccination and T1D onset. It does not show a distinctive time trend in the T1D onset after the introduction of COVID-19 vaccination as reported elsewhere [20]. This may also be because the intervention uptake was suboptimal even in the 12–17 age group and spanning over a 3-year period. Moreover, the unknown pathophysiology of how vaccination affects the outcome (diabetes onset) makes determining the optimal timeframe for measuring the expected outcome challenging. Nevertheless, for the first time, we reported the incidence of T1D in the 0–17-year age group during the whole pandemic and real-world data about COVID-19 vaccination uptake.

In conclusion, the incidence of T1D in children aged 0–17 years remained stable during the COVID-19 pandemic, regardless of the COVID-19 vaccination uptake. Post-COVID surveillance studies are needed to evaluate the incidence trend of T1D in the following years. Moreover, further research is needed to explore why, at disease onset, the vaccination uptake among diabetes onset was lower than the general population despite equal opportunities to access immunization services.
